# Chemical Composition, Antifungal, and Cytotoxicity Activities of* Inga laurina* (Sw.) Willd Leaves

**DOI:** 10.1155/2019/9423658

**Published:** 2019-02-03

**Authors:** Carla de Moura Martins, Sérgio A. L. de Morais, Mário M. Martins, Luís C. S. Cunha, Cláudio V. da Silva, Carlos H. G. Martins, Luís F. Leandro, Alberto de Oliveira, Francisco J. T. de Aquino, Evandro A. do Nascimento, Roberto Chang

**Affiliations:** ^1^Chemistry Nucleus, Goiano Federal Institute, Campus Morrinhos, BR-153, km 633, Rural Area, 75650-000 Morrinhos-GO, Brazil; ^2^Natural Products Research Nucleus (NuPPeN), Federal University of Uberlândia, João Naves de Ávila Avenue, 2121, Santa Mônica, 38400-902 Uberlândia-MG, Brazil; ^3^Institute of Biomedical Sciences, Laboratory of Trypanosomatids, Federal University of Uberlândia, Pará Avenue, 1720, Umuarama, 38405-320 Uberlândia-MG, Brazil; ^4^Laboratory of Research on Applied Microbiology, Franca University (UNIFRAN), Dr. Armando Salles Oliveira Avenue, 201, University Park, 14404-600 Franca-SP, Brazil

## Abstract

The species* Inga laurina* is native to the Brazilian Cerrado. There are no studies about the chemical composition and biological activities of extracts of this endangered species. The ethanolic extract and its successive fractions are rich in phenolic compounds and presented good antifungal activities. HPLC/MS-MS/MS and H1/C13 analysis led to the identification of seventeen compounds, most of which are gallic acid derivatives, myricetin and quercetin glycosides. The ethyl acetate fraction (EAF) contained high levels of total phenolics, expressed in milligrams of gallic acid equivalents per gram of extract (475.3 ± 1.9 mg GAE g_extract_^−1^) and flavonoids expressed in milligrams of quercetin equivalents per gram of extract (359.3 ± 10.6 mg QE g_extract_^−1^). This fraction was active against fungi of the* Candida* genus. The EAF showed MIC value 11.7 *μ*g mL^−1^ against* C. glabrata *and a selectivity index of 1.6 against Vero cells. The flavonol glycoside myricetin-3-O-rhamnoside was isolated for the first time from the* Inga laurina*. These results make* I. laurina* a promising plant as a source of pharmaceutical and biological active antifungal compounds.

## 1. Introduction

Among the plants in danger of extinction from Brazilian savannah biome (Cerrado), the* In*ga spp. (Fabaceae) are found mainly in neotropical areas and many plants from this family have been reported to possess biological activities and several are used in folk medicine [1]. Leaves and roots of* I. strigillosa* are used by the indigenous tribes of the Amazon region for skin wounds, the flowers of* I. cecropietorum* are used for earache, and the flowers of* I. rubiginosa* are used against nasal congestion [2].

There are 131 species of this genus in Brazil, with 51 of them being endemic [3]. Several biological activities have been observed in* Inga* species, such as the antioxidant activity of* I. edulis *[4–6] and* I. verna *[2], the antifungal activity of* I. marginata *[7], the antimicrobial activity of* I. fendleriana *[8], the allelochemical effects in* I. umbellifera* [9], and the antitumoral activity in* I. marginata *[10].

The sp.* I. laurina* is found not only in the Cerrado biome but in different regions of Brazil: Amazon, Caatinga, and the Atlantic Coast. This plant is easily found in Brazil, but there are a few studies about its essential oil. The present work is based on a doctorate thesis and the aim of the present work was to identify the bioactive metabolites by Mass Spectrometry and to evaluate the antifungal activity against* Candida* genus and the cytotoxic activity on Vero cells ATCC CCL 81 using ethanolic extract and fractions of* I. laurina* leaves.

A few studies of plants from the Brazilian savannah have identified bioactive metabolites by Mass Spectrometry. The leaves of the plant* Banisteriopsis laevifolia* (A. Juss.) B. Gates presented mainly phenolic compounds, as flavonoid glycosides of quercetin derivatives [11].

## 2. Materials and Methods

### 2.1. Plant Material

Leaf samples of* I. laurina* (Sw.) Willd, Fabaceae, were collected near the BR-050 in Uberlândia, Minas Gerais State, Brazil (18°59′13.96′′S; 48°12′42.16′′W) in the month of February (2012). The specimens were identified by Prof. Dr. Glein Monteiro de Araújo, from the Biology Institute, Federal University of Uberlândia. An exsiccate was deposited at Herbarium Uberlandense (HUFU), under number 64050.

### 2.2. Extracts Preparation

Leaves were dried at 35°C and then shredded in a knife mill. The extract was prepared by maceration at room temperature with ethanol (95%). The mass of 900.0 grams of leaves were extracted over 48 hours (3x). Filtrates were joined, concentrated under vacuum (under 40°C), and freeze-dried (Lyophilizer LS 3000, TERRONI, Brazil). A yield of 54.0 grams was obtained. The ethanolic extract (EE) was stored under refrigeration until analysis.

### 2.3. Liquid-Liquid Extraction

The EE (42.0 g) was redissolved in 570.0 mL of methanol:water (9:1) and subjected to liquid-liquid extraction using solvents of increasing polarities (hexane, chloroform, ethyl acetate, and* n*-butanol). Three extractions were performed using 300.0 mL of solvent per extraction. Fractions were concentrated to dryness giving hexane (12.2 g), chloroform (4.1 g), ethyl acetate (3.2 g), and* n*-butanol (7.4 g) fractions. These fractions were freeze-dried and stored under refrigeration until analysis.

### 2.4. Spectrophotometric Analysis of Total Phenolics, Proanthocyanidins, and Flavonoids Contents

Total phenolics were determined by the Folin-Ciocalteu method described by Morais et al. [29] and the results were expressed in mg of gallic acid equivalents by gram of dry extract (mg GAE g_extract_^−1^). Proanthocyanidin content was determined by the sulfuric vanillin method according to Morais et al. [29] and expressed as milligrams of catechin equivalents per gram of dry extract (mg CE g_extract_^−1^). Flavonoid content was determined as described by Woisky and Salatino [30] and expressed in mg of quercetin equivalents per gram of dry extract (mg QE g_extract_^−1^). The readings were taken in a Thermo Scientific Genesys-10S spectrophotometer. All analyses were performed in triplicate.

### 2.5. Antifungal Activity

#### 2.5.1. Microbial Strains and Minimum Inhibitory Concentration (MIC)

The following microorganisms obtained from the American Type Culture Collection (ATCC, Rockville MD, USA) were used:* Candida albicans* (ATCC 28366),* Candida tropicalis* (ATCC 13803), and* Candida glabata* (ATCC 15126). The MIC determination for antifungal assay was performed according to the CLSI (Clinical and Laboratory Standard Institute) using the broth dilution assay method [31]. This assay way followed the methods of Nunes et al. 2016 [11]. The Amphotericin B and strains ATCC 22019 (*Candida parapsilosis*) and ATCC 6258 (*Candida krusei*) were used as quality controls.

### 2.6. Cytotoxicity Assay (CC_50_) Using Vero Cells

The cell viability test was performed with Vero cells (ATCC CCL 81) (kidney fibroblasts, African green monkey). The cytotoxic activity was performed using the microplate dilution method [32]. Cell viability was calculated from the absorbance of each concentration tested according to the growth control. The cytotoxic concentration (CC_50_) (concentration that presents 50% cell viability) was calculated by means of a dose-response graph with nonlinear regression [33]. Controls of growth, solvent, samples, negative control (100% lysed cells), and control of the medium were performed. The assays were performed in triplicate. The selectivity index (SI) of each sample was defined as the ratio between the logarithm of CC_50_ and the MIC against each strain (SI = log[CC50]/[MIC]) [34, 35].

### 2.7. Ethyl Acetate Fraction (EAF) Isolation and Fractionation

Due to the fact that EAF of* I. laurina* showed promising results for inhibiting antifungal growth, it was selected for refractionation. Thus, a sample of EAF (0.60 g) was submitted to Column Chromatography with Sephadex LH-20 (34.0 g, 30.0 × 3.0 cm). The column was eluted with ethyl acetate:methanol in a stepwise gradient (300.0 mL 90:10; 300.0 mL 80:20; 300.0 mL 70:30; 200.0 mL 60:40; 200.0 mL 50:50; 200.0 mL 40:60; 200.0 mL 30:70; 100.0 mL 20:80; 100.0 mL 10:90). At the end, the column was washed with 300.0 mL of MeOH. The collected fraction volume was 15.0 mL and fractions with the same profile on TLC chromatograms were joined together. The fractionation of EAF yielded 8 subfractions (F1 to F8). Fractionation was repeated with another 0.60 g of EAF to obtain a higher mass of fraction. Both fractionations yielded about 500.0 mg of pure myricetin-3-O-rhamnoside in fraction 4 after TCL monitoring.

Myricetin-3-*O*-rhamnoside: M.p.: decomposes near 200°C. UV (MeOH): *λ*_max_ 258 and 353 nm; ^1^H NMR (DMSO-*d*_6_, 400 MHz): *δ* 6.20 (1H,* d*,* J* = 2.0 Hz, H-6), 6.35 (1H,* d*,* J* = 2.0 Hz, H-8), 6.89 (2H,* s*, H-2′e H-6′), 5.20 (1H,* d*,* J* 1.2 Hz, H-1′′), 3.98 (1H,* s*, H-2′′), 3.55 (1H,* dd*,* J* 1.2 e 9.3 Hz, H-3′′), 3.16 (1H,* m*, H-4′′), 3.37 (1H,* m*, H-5′′), 0.84 (3H,* d*,* J* 6.2 Hz, H-6′′); ^13^C NMR (DMSO-*d*_6_, 100 MHz,): *δ* 156.4 (C-2), 136.5 (C-3), 177.8 (C-4), 161.3 (C-5), 98.7 (C-6), 164.2 (C-7), 93.5 (C-8), 157.5 (C-9), 104,1 (C-10), 119.6 (C-1′), 107.9 (C-2′), 145.8 (C-3′), 134.3 (C-4′), 145.8 (C-5′), 107.9 (C-6′), 101.9 (C-1′′), 70.0 (C-2′′), 70.4 (C-3′′), 71.3 (C-4′′), 70.6 (C-5′′), 17.5 (C-6′′). HPLC-ESI/MS^2^* m/z*: 463.0889 ([M – H]^–^, C_21_H_19_O_12_^–^ calc. 463.0882).

### 2.8. High Performance Liquid Chromatography–Electrospray Ionization–Tandem Mass Spectrometry (HPLC-ESI/MS^n^)

The assays were carried out in a Liquid Chromatography system (Agilent Infinity 1260) coupled to a High Resolution Mass Spectrometer with Quadrupole Time of Flight (QTOF) (Agilent® model 6520 B) with an Electrospray Ionization source (ESI). The chromatographic parameters were Agilent Zorbax column, 2.1 mm internal diameter, 5 cm long, 1.8 *μ*m particles, mobile phase: water acidified with formic acid (0.1%, v v^−1^) (A) and methanol (B), with the following solvent gradient system: 2% B (0 min), 98% B (0-15 min); 100% B (15-17 min); 2% B (17-22 min). Nitrogen (N_2_) was used as a drying gas at a flow 8 L min^−1^ and as a nebulizing gas at a pressure of 58 psi. The nebulizer temperature was set at 220°C with a potential of 4.5 kV used on the capillary.

### 2.9. Nuclear Magnetic Resonance (NMR)

NMR spectra were carried out in the Bruker Model Ascend™ 400 Avance III HD (9.2 Tesla) spectrometer. The samples were solubilized in deuterated dimethyl sulfoxide (DMSO-d_6_) and tetramethylsilane (TMS) was used as an internal standard. Analyses were performed at 400 MHz for ^1^H NMR and at 100 MHz for ^13^C NMR. The following NMR analyses were performed: ^1^H, ^13^C, DEPT-135, COSY, and HSQC.

### 2.10. High Performance Liquid Chromatography (HPLC)

The EAF and subfractions from the leaves were analyzed by High Performance Liquid Chromatography coupled to a diode array detector (HPLC-DAD). A Shimadzu Chromatography system, model LC-6AD with C18 reverse phase column (Phenomenex Luna model, 4.6 mm internal diameter, 25 cm long, 5 *μ*m particles with 100 Å diameter), was used. A volume of 20 *μ*L of methanol solution was injected at 3,000 *μ*g mL^−1^ of EAF and 1,000 *μ*g mL^−1^ of subfraction (F1-F8). Deionized water (phase A) and methanol (phase B) were used as mobile phases using the following program: 50% B (25 min) at a flow rate of 0.8 ml min^−1^.

### 2.11. Statistical Analysis

The analyses were performed in triplicate and the results were evaluated using the Analysis of Variance (ANOVA) method. The results were considered statistically different when the significance level was lower than 5% (P<0.05). The Tukey test was used to determine the significant differences between the averages. Analyses were performed using the SigmaPlot 11.0 program.

## 3. Results and Discussion

### 3.1. Phenolics, Proanthocyanidins, and Flavonoids Contents

Spectrophotometric results for extracts and fractions of* I. laurina* leaves are shown in Table 1.

EE, EAF, and BF fractions presented the highest contents of total phenolics. The difference in these values can be explained by the polarity of the solvents used [6]. Polar solvents such as ethanol, ethyl acetate, and* n*-butanol are more able to extract phenolic compounds. EE and EAF have higher levels of total phenolics when compared to those of* I. marginata*, which has values of 31.63 mg and 8.37 of GAE g_extract_^−1^, respectively [36]. The methanolic extract (50%) of* I. edulis *presented total phenolics of 496.5 mg of GAE g_extract_^−1^, which is higher than that of EE from* I. laurina* [4]. Dias, Souza, and Rogez [6] reported values of 15.8 and 357.5 mg GAE g_extract_^−1^ of total phenolics for hexane and water fractions, respectively, for the acetone:water:acetic acid extract (70:28:2 v:v:v). The values of proanthocyanidins were lower when compared with total phenolics. No data were found for this technique in the literature concerning levels of proanthocyanidins for* I.* species.

The fractions CF and EAF presented the highest values of flavonoids among the other fractions. The good result obtained with the less polar fraction (chloroform) suggests the presence of aglycone flavonoids. Flavonols and flavones were confirmed by the test of complexation with aluminum ions (Al^3+^). This complexation reaction allows the quantification of flavonoids by reading the absorbance of the solution. In this reaction, the aluminum chloride shifts the wavelengths of bands I and II to a higher wavelength, a bathochromic shift [37]. EE has higher level of flavonoids when compared to that presented by* I. marginata*, whose value is 118 mg QE gextract^−1^ [38].

### 3.2. Antifungal and Cytotoxicity Activities

Values of MIC (minimum inhibitory concentration) for antifungal activity in *μ*g mL^−1^ and cytotoxic activity in CC_50_ (cytotoxic concentration), in *μ*g mL^−1^, for extract and fractions of leaves of* I. laurina* are shown in Table 2.

Fractions EE, EAF, and BF were the most active against the evaluated microorganism, showing MIC values lower than 100 *μ*g mL^−1^, as shown in Table 2. In general, the BF fraction showed good activity against* C. albicans* (MIC 11.7 *μ*g mL^−1^) and for* C. glabrata* (MIC 23.4 *μ*g mL^−1^); the EE and EAF fractions were also very effective against* C. glabrata *(MIC 11.7 *μ*g mL^−1^); and the CF fraction had no antifungal activity at the tested concentrations (MIC above 3,000 *μ*g mL^−1^). The EE of the leaves from* B. laevifolia* showed good results for antifungal activity, showing MIC values of 31, 63, and 63 *μ*g mL^−1^ for* C. albicans, C. tropicalis, *and* C. glabrata*, respectively [11].

Regarding the cytotoxic activity, the lower the CC_50_ value is, the higher the cytotoxicity against Vero cells will be, because a small concentration of the sample would inhibit the growth of cells by 50%. To correlate the antifungal activity with the cytotoxic concentration, the selectivity index (SI) was calculated. The SI indicates whether the sample is more selective for antifungal activity or more toxic for Vero cells. The more positive the SI value, the greater the selectivity to inhibit fungal growth; a negative value indicates that the sample is more toxic to Vero cells than selective for the inhibition of antimicrobial growth [34]. Therefore, the fractions EE, EAF, and BF were the most selective to the tested microorganisms, as they presented positive SI values for all of the fungi of the genus* Candida*; the best values were 1.5, 1.6, and 1.6, respectively. Nunes et al. [11] also found positive SI values for EE and* n*-butanol partition from* B. laevifolia* leaves, for the same microorganisms tested in the Table 2. The best results were found for E, which showed a positive SI value of 1.2, 0.9, and 0.9 for* C. albicans, C. tropicalis, *and* C. glabrata*, respectively.

Other* Inga* species were studied regarding antifungal activity. The EE of leaves of* I. vera* showed low inhibition against* C. albicans*, with an inhibition zone of 7 to 15 mm (i.d.), whereas a good inhibition would be larger than 20 mm [39]. The ethanolic extract of* I. marginata* leaves showed no antifungal activity against* C. albicans *and* C. krusei* but showed activity against* C. tropicalis* [40]. The promising antifungal activity of* I. laurina* can be related to the presence of phenolic compounds. The extracts and fractions showed a high quantity of total phenolics and flavonoids and these compounds have been frequently reported as potential antifungal agents [41–45].

### 3.3. High Performance Liquid Chromatography–Electrospray Ionization–Tandem Mass Spectrometry (HPLC-ESI/MS^2^) Analysis

**Figure 1:**
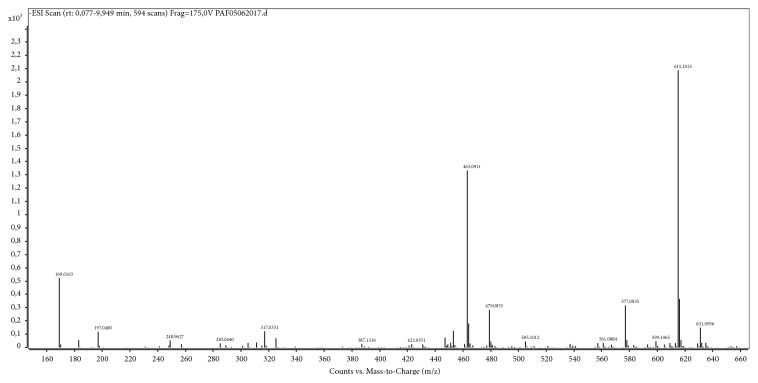
Total ion chromatogram of EAF obtained by HPLC-ESI.

**Figure 2:**
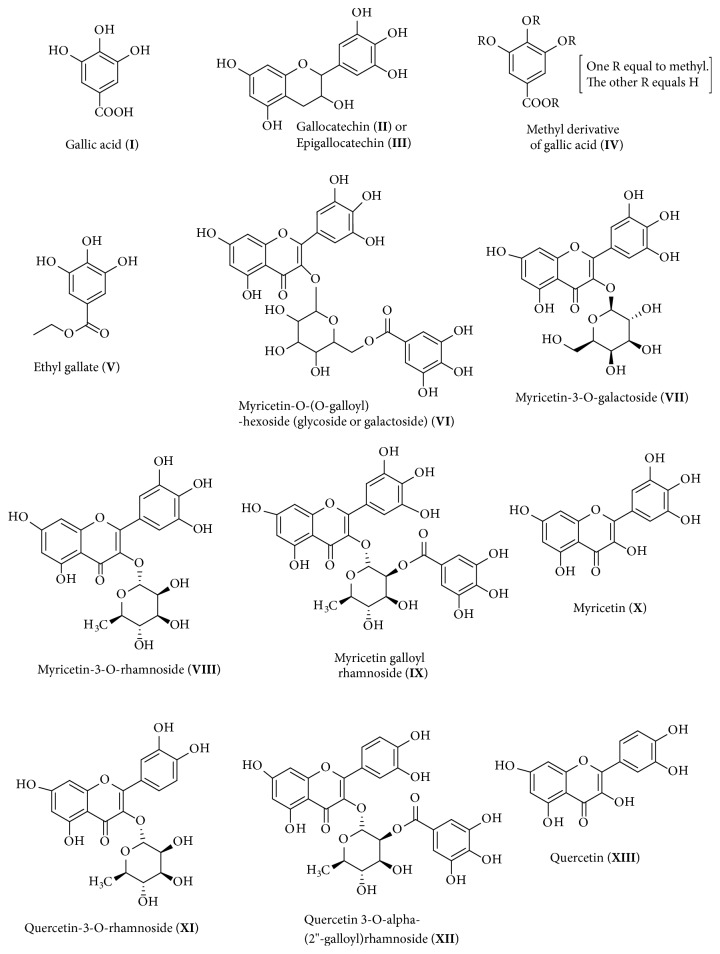
Compounds identified in the EAF.

The EAF presented the highest level of phenolic compounds and flavonoids compared to the other fractions and showed good results for antifungal activity. Therefore, this polar fraction was submitted to High Performance Liquid Chromatography coupled to Electrospray Ionization (HPLC-ESI) analysis and sequential Mass Spectrometry (MS/MS) in the negative mode. The total ion chromatogram of EAF obtained by HPLC-ESI is shown in Figure 1. Thirteen compounds could be identified in this fraction using this technique (Table 3 and Figure 2).

**Figure 3:**
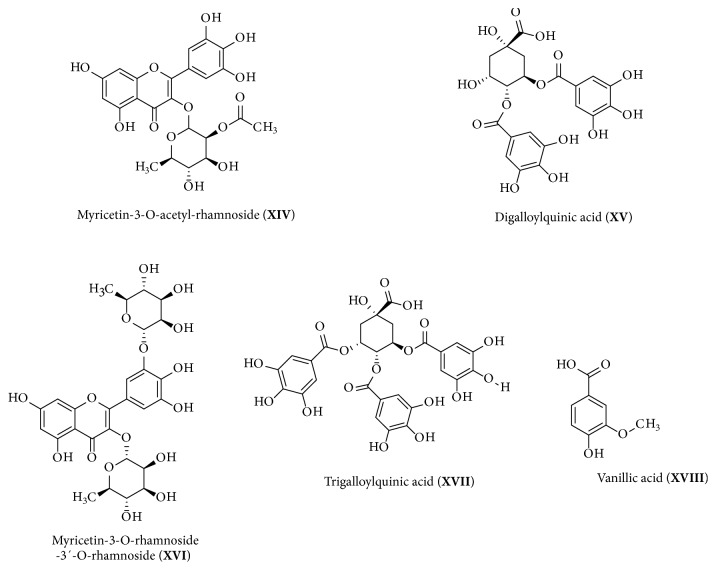
Compounds identified in subfractions F2-F7.

Column fractions (F1-F8) obtained from EAF were also analyzed by HPLC-ESI/MS^2^. Five different compounds were identified in addition to those identified in EAF (Table 3); their structures are shown in Figure 3. The compound myricetin-3-*O*-acetyl-rhamnoside (compound** XIV**) was identified in fraction F2 (*m/z* 505), digalloylquinic acid (compound** XV**,* m/z* 495) and myricetin-3-*O*-rhamnose-3′-*O*-rhamnoside (compound** XVI**,* m/z* 609) were identified in F5, trigalloylquinic acid (compound** XVII,*** m/z* 647) was identified in the F6, and vanillic acid (compound** XVIII**,* m/z* 167) was identified in F7 (Table 3). See Scheme S1 in the Supplementary Material for the comprehensive flowchart of purification, identification, and isolation of compounds from the EAF.

**Figure 4:**
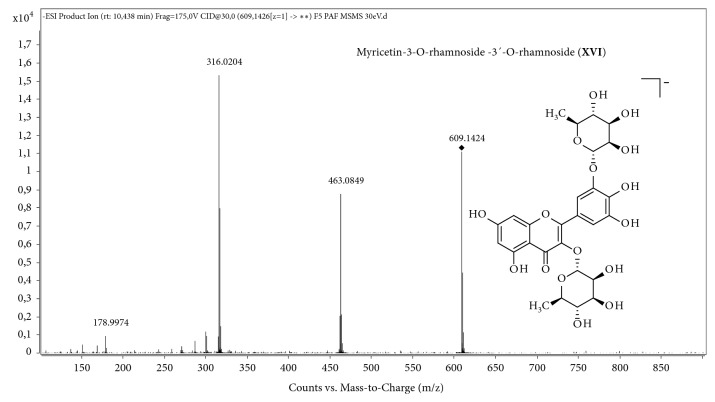
MS/MS spectrum of ion* m/z* 609.

The ion* m/z* 609 (compound** XVI**) forms the fragments* m/z* 463, 316, and 178 (Figure 4), which correspond to compound** VIII**, which was confirmed to be myricetin-3-*O*-rhamnoside (Figure 2). The loss of 146 Da (rhamnosyl group) of ion* m/z* 609 forms the ion* m/z* 463, which loses a further 146 Da to form the ion* m/z* 316. This finding is in accordance with the structure proposed for the ion* m/*z 609, which has 2 rhamnosyl groups bonded to the myricetin aglycone. The literature reports a structure for myricetin-3′-*O*-rhamnose-3-*O*-galactoside [46]; therefore, a similar structure carrying two rhamnosyl groups in positions 3 and 3′ of the aglycone was proposed: myricetin-3-*O*-rhamnose-3′-*O*-rhamnoside. No reports on the identification of compounds II, III, VI, VII, IX, XII, XIV, XV, XVI, or XVII in the* Inga* species have been found.

The Figures S1to S16 show mass spectrum of phenolic compounds acquired by HPLC-ESI/MS^2^. Figures S17to S22 show fragmentation patterns.

### 3.4. Structural Determination and Characterization of the Isolated Compound from Fraction F4 (Myricetin-3-O-Rhamnoside)

Fraction F4 was also analyzed by HPLC to check its purity. The HPLC chromatogram indicated the presence of only one intense peak in 8.1 min, while the UV/Vis spectrum shows two bands of absorption, characteristic for flavonoids (258 and 353 nm). See Figure S23 in the Supplementary Material to check the chromatogram and UV/Vis spectrum of F4.

The high resolution mass spectrum and NMR spectra confirmed the structure of the isolated compound in fraction 4 as myricetin-3-*O*-rhamnoside, a glycosylated flavonoid with crystalline aspects, with the molecular formula C_21_H_20_O_12_ and MW 464.38 gmol^−1^ (compound** VIII**, Figure 2). There are no records for its melting point in the literature, but it decomposes close to 200°C and its color changes from yellow to orange and black.

**Figure 5:**
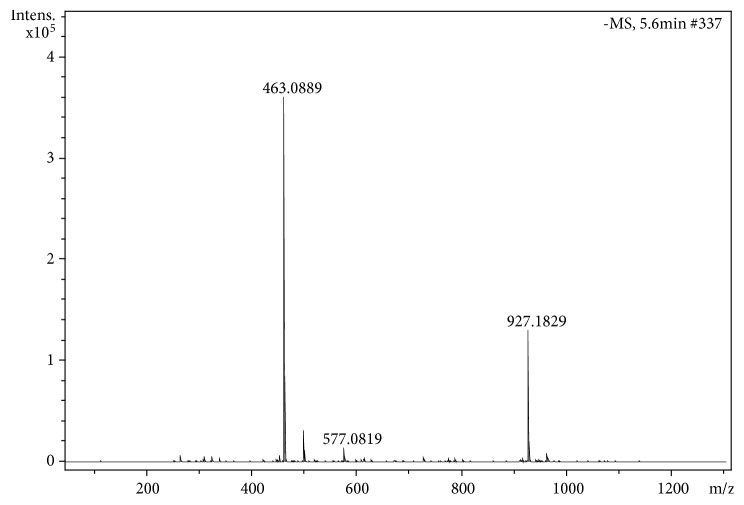
High resolution mass spectra of fraction F4.

Figure 5 shows the high resolution mass spectrum of F4; it is possible to verify the molecular weight of the compound [M – H]^−^ at ion* m/z* 463.0889 (acquired mass). The exact mass of compound** VIII** was* m/z* 463.0882 (C_21_H_19_O_12_)^−^, with an error of 1.5 ppm. The peak at* m/z* 927.1829 corresponds to the ion* cluster* formation of** VIII**.

**Figure 6:**
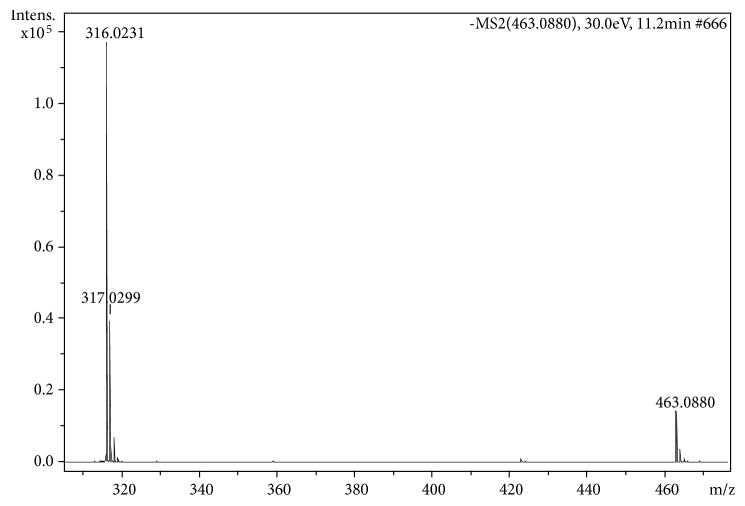
MS/MS spectrum of the ion* m/z* 463.

The main fragmentation of VIII is the loss of the rhamnoside radical, yielding the ion radical at* m/z* 316 [M^−^ – 147]^−^; with lower intensity, the aglycone can be produced (M^−^ –146]^−^ after the loss of an unsaturated rhamnoside of mass 146 Da (Figure 6). The spectrum in Figure 5 is in good agreement with Saldanha et al. [17], where flavonoids in* Myrcia bella* Cambess were identified by using the same technique as in the present work (MS/ESI-MS/MS). The fragmentation pathway of myricetin-3-*O*-ramnoside corresponds to that proposed by [19] when they studied the methanol extract of* Pistacia lentiscus* leaves.

^1^H NMR spectra showed 3 different signals attributed to aromatic hydrogens, two doublets were observed at *δ* 6.20 and *δ* 6.37, both with* J* = 2.0 Hz, typical of meta -couplings of carbons C-6 and C-8, respectively, in ring A. There is an intense singlet in *δ* 6.89 attributed to hydrogens H-2′ and H-6′ of ring B, which are equivalent. The signal at *δ* 12.00 corresponds to the hydroxyl hydrogen of carbonyl C-5, which has a hydrogen bond with a carbonyl carbon C-4, corresponding to a chelated hydroxyl.

**Figure 7:**
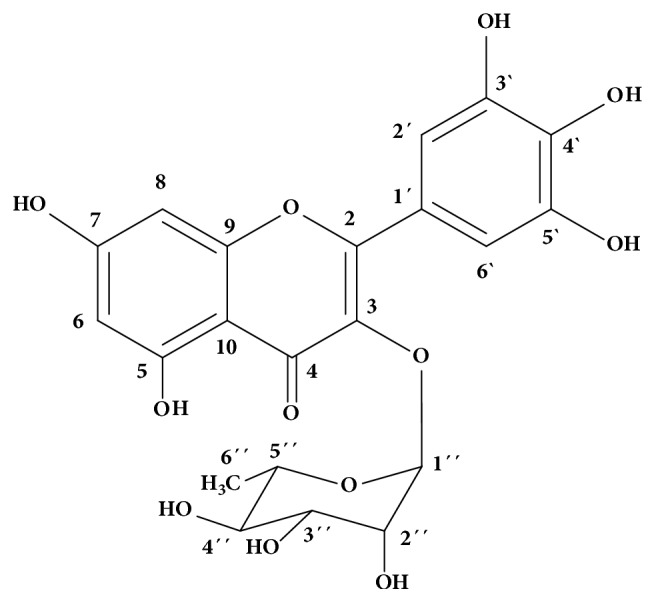
Structure of myricetin-3-O-rhamnoside compound isolated from the fraction F4.

In HSQC contour map analysis, it was possible to observe a signal at *δ* 5.2 (1H,* d*,* J* = 1.2 Hz), typical of a hydrogen bound to the anomeric carbon (*δ* 101.94, C-1′′) of rhamnose. This coupling constant value is typical of hydrogen in the equatorial position of the glycosidic ring which is coupled with the hydrogen H-2′′ (axial-equatorial or equatorial-equatorial). The DEPT-135 and ^13^C NMR spectra, respectively, allowed confirmation of the carbon signals in the aromatic region. The correlations of the hydrogens of rhamnose were attributed through the COSY spectrum. Thus, it can be inferred that the isolated compound is myricetin-3-*O*-rhamnoside [4, 47] (Figure 7). See Figures S24–S29 and Tables S1–S2 in the Supplementary Material to check the NMR spectra and NMR chemical shifts, respectively, of F4.

Myricetin-3-*O*-rhamnoside inhibited the growth of all yeasts as shown in Table 2 at a concentration of 93.8 *μ*g mL^−1^. This result confirms the antifungal activity for this compound. Salazar-Aranda and coworkers [48] also observed an MIC higher than 83 *μ*g mL^−1^ for* C. albicans* and* C. tropicalis*, although the MIC values were higher for* C. glabrata*, 3.9 *μ*g mL^−1^, and 7.8 *μ*g mL^−1^ for clinical isolates of yeast.

The isolated compound presents the same MIC of EAF for the evaluated microorganism, except for* C. glabrata*, which has a lower value (11.7 *μ*g mL^−^). From these results, it is possible to infer that myricetin-3-*O*-rhamnoside can contribute markedly to the antifungal activity observed for EAF against* C. albicans* and* C. tropicalis*, since they presented the same MIC values.

## 4. Conclusions

The EE from the leaves of* Inga laurina* and its fractions are rich in phenolic compounds and present good antifungal activities. HPLC/MS-MS/MS and H1/C13 analysis made it possible to identify seventeen compounds, most of which are gallic acid derivatives and myricetin and quercetin glycosides. The EAF contained a high level of total phenolics (475.3 ± 1.9 mg GAE g_extract_^−1^) and flavonoids (359.3 ± 10.6 mg QE g_extract_^−1^) and was active against fungus from the* Candida* genus. The best MIC results found for* C. albicans, C. glabrata, *and* C. tropicalis* were 11.7, 11.7, and 46.8 *μ*g mL^−1^, respectively, and the best selectivity indexes for the three microorganisms, using Vero cells, were 1.6, 1.3, and 1.0, respectively. These results make* I. laurina* a promising plant for advanced antifungal studies.

## Figures and Tables

**Table 1:** Total phenolics, proanthocyanidins, and flavonoids for extracts and fractions of *I. laurina* leaves.

Samples	TP (mg GAE g_extract_^−1^)	P (mg CE g_extract_^−1^)	F (mg QE g_extrac_t^−1^)
EE	127.7 ± 0.1	76.7 ± 1.3^a^	133.1 ± 3.5
HF	35.0 ± 0.1	20.2 ± 1.9	33.6 ± 2.1^a^
CF	82.2 ± 1.0	43.3 ± 4.5	205.9 ± 9.2
EAF	475.3 ± 1.9	68.1 ± 4.4^a^	359.3 ± 10.6
BF	135.9 ± 1.5	59.7 ± 4.1	24.2 ± 1.2^a^

EE = ethanol extract; HF = hexane fraction; CF = chloroform fraction; EAF = ethyl acetate fraction; BF = *n*-butanol fraction; TP = total phenolics; GAE = gallic acid equivalent; P = proanthocyanidins; CE = catechin equivalent; F = flavonoids; QE = quercetin equivalent. Results are presented as mean ± standard deviation for the triplicate assays. The analyses with the same letters did not show a significant difference between the averages by 5% Tukey test.

**Table 2:** Results of antifungal activity (expressed as MIC in *μ*g mL^−1^), cytotoxic activity (expressed as CC_50_ in *μ*g mL^−1^), and selectivity index for extract and fractions of *I. laurina *leaves.

	**MIC (**μ**g mL**^**−1**^**)**				

Microorganisms	EE	HF	CF	EAF	BF

*C. albicans *ATCC 28366	46.8	1,500	>3,000	93.8	11.7
*C. glabrata *ATCC 15126	11.7	187.5	>3,000	11.7	23.4
*C. tropicalis *ATCC 13803	93.8	3,000	>3,000	93.8	46.8

	**CC**_**50**_ ** (**μ**g mL**^**−1**^**)**				

Vero cells	352 ± 5	384 ± 9	>512	>512	>512

	**SI**				

Microorganisms	EE	HF	CF	EAF	BF

*C. albicans *ATCC 28366	0.9	- 0.6	> - 0.8	> 0.7	> 1.6
*C. glabrata *ATCC 15126	1.5	0.3	> - 0.8	> 1.6	> 1.3
*C. tropicalis *ATCC 13803	0.6	- 0.9	> - 0.8	> 0.7	> 1.0

EE = ethanol extract; HF = hexane fraction; CP = chloroform fraction; EAF = ethyl acetate fraction; BF = *n*-butanol fraction. SI: selectivity index. ATCC: American Type Culture Collection.

**Table 3:** Phenolic compounds identified in EAF and subfractions from *I. laurina* by HPLC-ESI/MS^2^.

**Fractions/Subfractions**	t_R(min)_	**[M – H]** ^–^	**Exact ** **mass**	**Error (ppm)**	**Fragments** ***m/z***	**Molecular formula **	**Compound**	**References**
EAF	2.9	169.0154	169.0142	-7.1	125	C_7_H_6_O_5_	Gallic acid (**I**)	[12, 13]
	5.4	305.0663	305.0667	1.3	261, 219, 167/165, 125	C_15_H_14_O_7_	Gallocatechin (**II**) or Epigallocatechin (**III**)	[14, 15]
	6.9	183.0300	183.0299	-0.5	168, 124	C_8_H_8_O_5_	Methyl derivative of gallic acid (**IV**)	[12]
	9.1	197.0468	197.0455	-6.6	169, 124/123	C_9_H_10_O_5_	Ethyl gallate (**V**)	[14, 16]
	10.1	631.0956	631.0941	-2.4	479, 316, 169	C_28_H_24_O_17_	Myricetin-*O*-(*O*-galloyl)-hexoside (glycoside or galactoside) (**VI**)	[17, 18]
	10.7	479.0826	479.0831	1.0	316, 178	C_21_H_19_O_13_	Myricetin-3-*O*-galactoside (**VII**)	[17, 18]
	11.1	463.0904	463.0888	-3.5	316, 178	C_21_H_19_O_12_	Myricetin-3-*O*-rhamnoside (**VIII**)	[17]
	12.2	615.1007	615.0992	-2.4	463, 316/317, 178	C_28_H_24_O_16_	Myricetin galloyl rhamnoside (**IX**)	[18, 19]
	12.4	317.0318	317.0303	-4.7	178, 151	C_15_H_10_O_8_	Myricetin (**X**)	[13, 14, 20]
	12.7	447.0955	447.0933	-4.9	300/301	C_21_H_20_O_11_	Quercetin-3-*O*-rhamnoside (**XI**)	[17, 18]
	13.5	599.1044	599.1042	-0.3	447, 301, 169, 151	C_28_H_24_O_15_	Quercetin 3-O-alpha-(2′′-galloyl)rhamnoside (**XII**)	[21, 22]
	13.8	301.0357	301.0354	-1.0	178/179, 151	C_15_H_10_O_7_	Quercetin (**XIII**)	[14]
F2	10.8	505.0999	505.0988	- 2.2	463, 316, 271, 163	C_23_H_22_O_13_	Myricetin-3-O-acetyl-rhamnoside (**XIV**)	[23, 24]
F5	7.0	495.0786	495.0780	- 1.2	343, 169	C_21_H_20_O_14_	Digalloylquinic acid (**XV**)	[25–27]
F5	10.3	609.1482	609.1461	- 3.4	463, 316, 178	C_27_H_30_O_16_	Myricetin-3-O- rhamnoside -3′-O-rhamnoside (**XVI**)	Structure proposed by the author
F6	7.7	647.0885	647.0890	0.8	495, 343, 325, 169	C_28_H_24_O_18_	Trigalloylquinic acid (**XVII**)	[26]
F7	8.3	167.0355	167.0350	- 3.0	108	C_8_H_8_O_4_	Vanillic acid (**XVIII**)	[28]

## Data Availability

The data used to support the findings of this study are available from the corresponding author upon request. All data in our manuscript is available for readers.

## Supplementary Materials

Supplementary Materials Supplementary Scheme S1: flowchart for purification, identification, and isolation of compounds from the leaves of* I. laurina. *Supplementary Figures S1–S12: mass spectrum of ion* m/z* of phenolic compounds identified in EAF from* I. laurina* by HPLC-ESI/MS^2^. Supplementary Figures S13–S16: mass spectrum of ion* m/z* of phenolic compounds identified in fractions F2, F5, F6, F7, respectively, from* I. laurina* by HPLC-ESI/MS^2^. Supplementary Figures S17–S22: Figures S17 and S18 show fragmentation patterns (*m/z* 609 and* m/z *463) and fragmentation mechanisms of compounds identified in EAF. Supplementary Figure S23: chromatogram and UV/Vis spectrum of fraction 4 (F4). Supplementary Figure S24: ^1^H NMR spectra (400 MHz, DMSO-*d*_6_) of myricetin-3-*O*-rhamnoside. Supplementary Figure S25: ^13^C NMR spectra (100 MHz, DMSO-*d*_6_) of myricetin-3-*O*-rhamnoside. Supplementary Figure S26: HSQC contour map (DMSO-*d*_6_) of myricetin-3-*O*-rhamnoside in the aromatic region. Supplementary Figure S27: COSY contour map (DMSO-*d*_6_) of myricetin-3-*O*-rhamnoside. Supplementary Figure S28: amplification of the COSY (400 MHz, DMSO-*d*_6_) contour map in the region of glycosidic hydrogens of myricetin-3-*O*-rhamnoside. Supplementary Figure S29: DEPT-135 spectra (100 MHz, DMSO-*d*_6_) of myricetin-3-*O*-rhamnoside. Supplementary Table S1: ^1^H NMR (400 MHz, DMSO-*d*_6_,) data of myricetin-3-*O*-rhamnoside. Supplementary Table S2: ^13^C NMR (100 MHz, DMSO-*d*_6_) data of myricetin-3-*O*-rhamnoside.
