# Supplementary material for: Chemical Composition, Antifungal, and Cytotoxicity Activities of Inga laurina (Sw.) Willd Leaves
Source: ScientificWorldJournal. 2019 Feb 3;2019:9423658. doi: 10.1155/2019/9423658 (PMC6377950; doi:10.1155/2019/9423658)
Supplement: Supplementary Materials — Supplementary Scheme S1: flowchart for purification, identification, and isolation of compounds from the leaves of I. laurina. Supplementary Figures S1–S12: mass spectrum of ion m/z of phenolic compounds identified in EAF from I. laurina by HPLC-ESI/MS2. Supplementary Figures S13–S16: mass spectrum of ion m/z of phenolic compounds identified in fractions F2, F5, F6, F7, respectively, from I. laurina by HPLC-ESI/MS2. Supplementary Figures S17–S22: Figures S17 and S18 show fragmentation patterns (m/z 609 and m/z 463) and fragmentation mechanisms of compounds identified in EAF. Supplementary Figure S23: chromatogram and UV/Vis spectrum of fraction 4 (F4). Supplementary Figure S24: 1H NMR spectra (400 MHz, DMSO-d6) of myricetin-3-O-rhamnoside. Supplementary Figure S25: 13C NMR spectra (100 MHz, DMSO-d6) of myricetin-3-O-rhamnoside. Supplementary Figure S26: HSQC contour map (DMSO-d6) of myricetin-3-O-rhamnoside in the aromatic region. Supplementary Figure S27: COSY contour map (DMSO-d6) of myricetin-3-O-rhamnoside. Supplementary Figure S28: amplification of the COSY (400 MHz, DMSO-d6) contour map in the region of glycosidic hydrogens of myricetin-3-O-rhamnoside. Supplementary Figure S29: DEPT-135 spectra (100 MHz, DMSO-d6) of myricetin-3-O-rhamnoside. Supplementary Table S1: 1H NMR (400 MHz, DMSO-d6,) data of myricetin-3-O-rhamnoside. Supplementary Table S2: 13C NMR (100 MHz, DMSO-d6) data of myricetin-3-O-rhamnoside. [file 9423658.f1.docx]

**Supplementary Material**

**Chemical composition and antifungal and cytotoxicity activities of *Inga laurina* (Sw.) Willd leaves**

Carla de Moura Martins,^1^ Sérgio A. L. de Morais,^2^ Mário M. Martins,^2^ Luís C. S. Cunha,^2^ Cláudio V. da Silva,^3^ Luiz F. Leandro,^4^ Carlos H. G. Martins,^4^ Alberto de Oliveira,^2^ Francisco J. T. de Aquino,^2^ Evandro A. do Nascimento^2^ and Roberto Chang^2^

^1^Chemistry Nucleus, Goiano Federal Institute-Campus Morrinhos, BR-153, km 633, Rural Area, 75650-000, Morrinhos-GO, Brazil.

^2^Natural Products Research Nucleus (NuPPeN), Federal University of Uberlândia, João Naves de Ávila Avenue, 2121, Santa Mônica, 38400-902, Uberlândia-MG, Brazil.

^3^Institute of Biomedical Sciences-Laboratory of Trypanosomatids, Federal University of Uberlândia, Pará Avenue, 1720, Umuarama, 38405-320, Uberlândia-MG, Brazil.

^4^Laboratory of Research on Applied Microbiology, Franca University (UNIFRAN), Dr. Armando Salles Oliveira Avenue, 201, University Park, 14404-600, Franca-SP, Brazil.

Correspondence should be addressed to Roberto Chang; [chang@ufu.br](mailto:chang@ufu.br).

All data in our manuscript is available for readers.


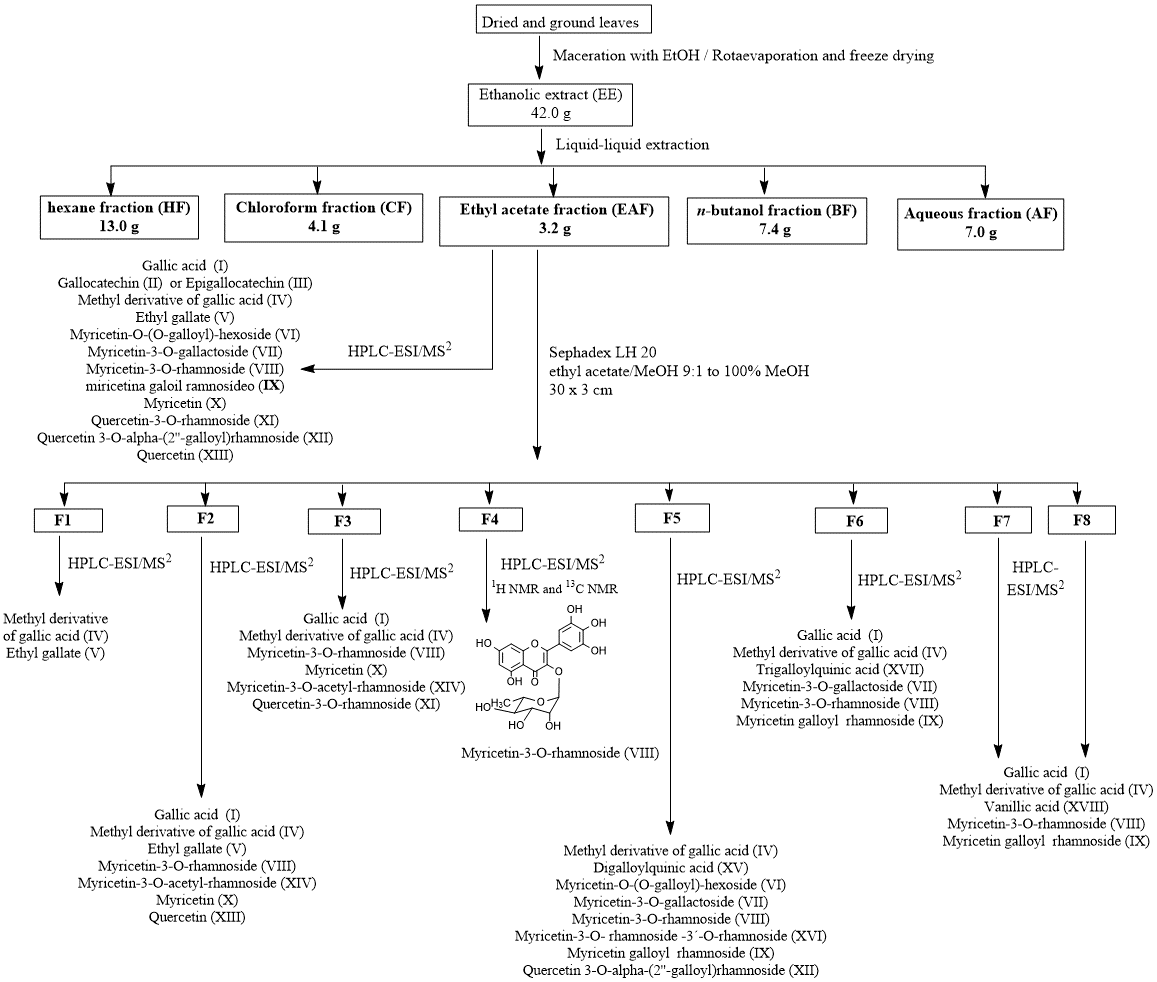


**­Scheme S1**:Flowchart for purification, identification and isolation of compounds from the leaves of *I. laurina*

**Figures S1 – S12**: Mass spectrum of ion *m/z* of phenolic compounds identified in EAF from *I. laurina* by HPLC-ESI/MS^2^

**Figure S1**: Mass spectrum of ion *m/z* 169 by MS/MS.

**Figure** **S2**: Mass spectrum of ion *m/z* 305 by MS/MS.

**Figure S3**: Mass spectrum of ion *m/z* 183 by MS/MS.

**Figure S****4**: Mass spectrum of ion *m/z* 197 by MS/MS.

**Figure S5**: Mass spectrum of ion *m/z* 631 by MS/MS.

**Figure S6**: Mass spectrum of ion *m/z* 479 by MS/MS.

**Figure S7**: Mass spectrum of ion *m/z* 463 by MS/MS.

**Figure S8**: Mass spectrum of ion *m/z* 615 by MS/MS.

**Figure S9**: Mass spectrum of ion *m/z* 317 by MS/MS.

**Figure S10**: Mass spectrum of ion *m/z* 447 by MS/MS.

**Figure S11**: Mass spectrum of ion *m/z* 599 by MS/MS.

**Figure S12**: Mass spectrum of ion *m/z* 301 by MS/MS.

**Figures S13–S16**: Mass spectrum of ion *m/z* of phenolic compounds identified in fractions F2, F5, F6, F7, respectively, from *I. laurina* by HPLC-ESI/MS^2^

**Figure S13**: MS/MS spectrum of ion *m/z* 505.

**Figure S14**: MS/MS spectrum of ion *m/z* 495.

**Figure S15**: MS/MS spectrum of ion *m/z* 647.

**Figure S16**: MS/MS spectrum of ion *m/z* 167.

**Figures S17–S22**: Fragmentation mechanisms of compounds identified in EAF.

**Figure S17.** Proposed mechanism for fragmentation of *m/z* 609 with loss of two rhamnoside structures.

**Figure S18**. Fragmentation mechanism for the ion *m/z* 463.

 **Figure S19**: Fragmentation mechanisms of compounds identified in EAF (**I-V**).

**Figure S20**: Fragmentation mechanisms of compounds identified in EAF (**VI-IX**).

**Figure S21**: Fragmentation mechanisms of compounds identified in EAF (**X-XIII**).

**Figure S22**: Fragmentation mechanisms of compounds identified in EAF (**XIV-XV**).

**Figure S23**: Chromatogram and UV/Vis spectrum of fraction 4 (F4).


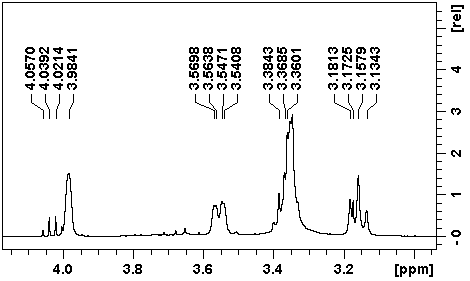

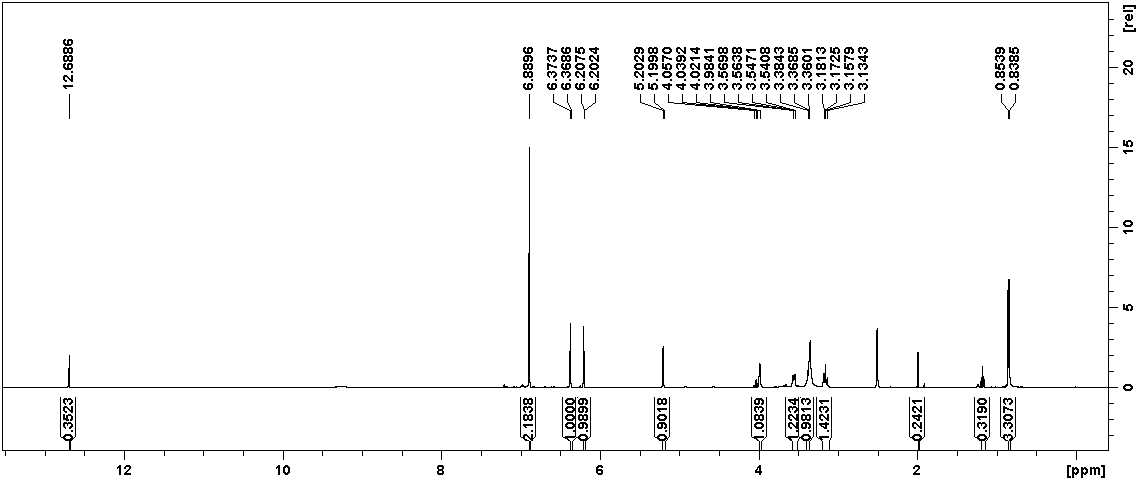


**6´´**

**4´´**

**3´´ 5´´**

**2´´**

**1´´**

**8; 6**

**2´;6´**

**Figure S24**: ^1^H NMR spectra (400 MHz, DMSO-*d*_6_) of myricetin-3-*O*-rhamnoside


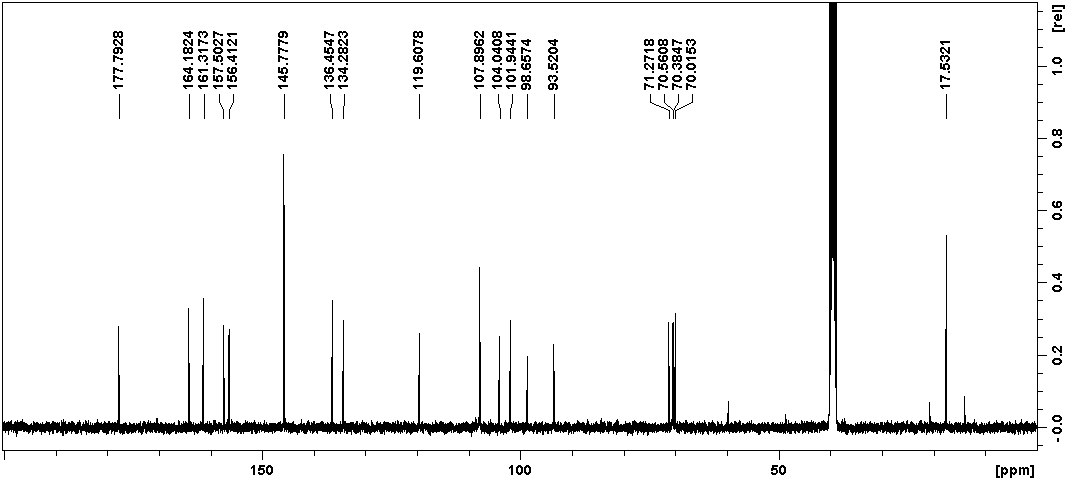

**Figure S25**: ^13^C NMR spectra (100 MHz, DMSO-*d*_6_) of myricetin-3-*O*-rhamnoside


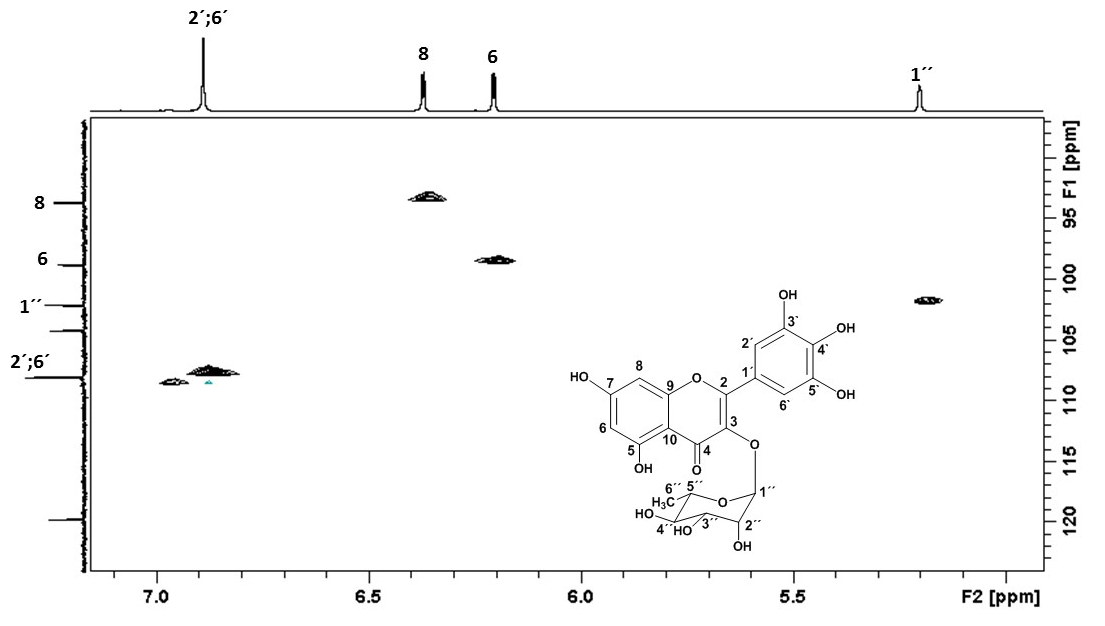


**Figure S26**: HSQC contour map (DMSO-*d*_6_) of myricetin-3-*O*-rhamnoside in the aromatic region.


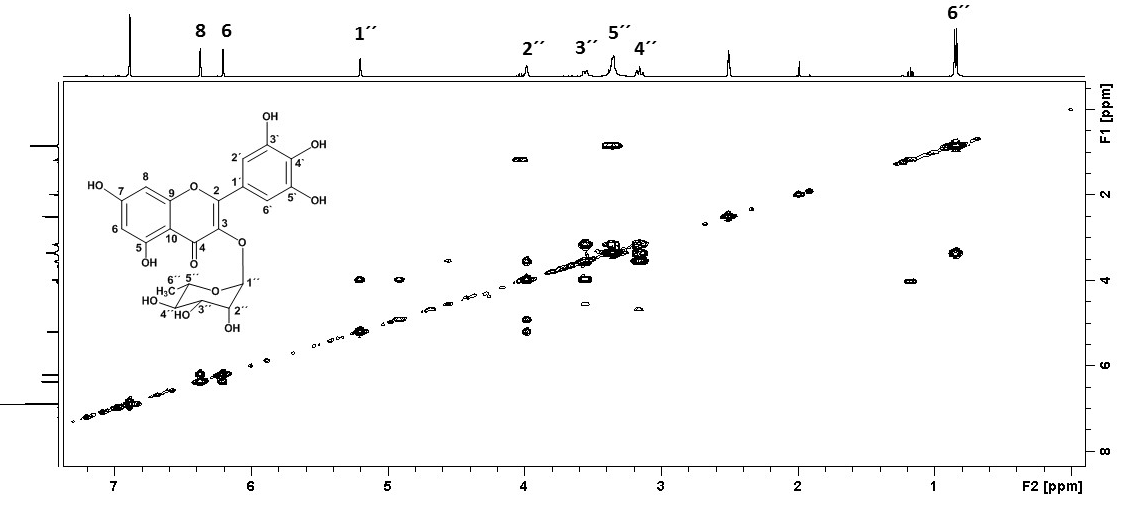


**Figure S27**: COSY contour map (DMSO-*d*_6_) of myricetin-3-*O*-rhamnoside.


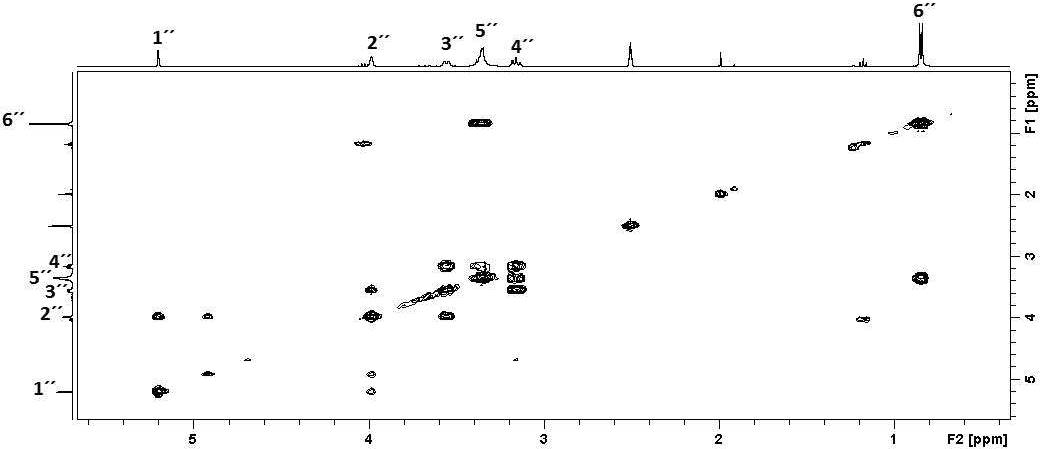


**Figure S28**: Amplification of the COSY (400 MHz, DMSO-*d*_6_) contour map in the region of glycosidic hydrogens of myricetin-3-*O*-rhamnoside.

**
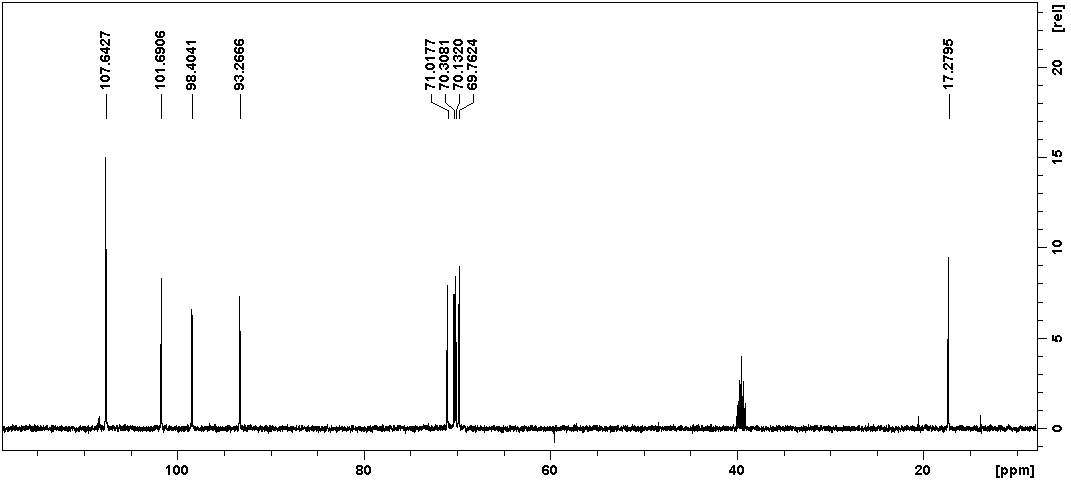
**

**6´´**

**2´´;3´´;5´´;4´´**

**2´;6´**

**1´´ 6 8**

**Figure S29**: DEPT-135 spectra (100 MHz, DMSO-*d*_6_) of myricetin-3-*O*-rhamnoside.

**Table S1**: ^1^H NMR (400 MHz, DMSO-*d*_6_,) data of myricetin-3-*O*-rhamnoside.

| **Position** | **^1^H NMR**  **(400 MHz,DMSO-*d*_6_)**  **δ^1^; m^2^; *J* ^3^** | **COSY** | **^1^H NMR**  **(300 MHz, DMSO-*d*_6_)**  **δ^1^; m^2^; *J*^3^**  ^[^[^1^](#_ENREF_1)^]^ |
| --- | --- | --- | --- |
| **6** | 6.20 d (2.0) | H-8 | 6.36 d (1.8) |
| **8** | 6.35 d (2.0) | H-6 | 6.18 d (1.8) |
| **2´** | 6.89 s | - | 7.04 s |
| **6´** | 6.89 s | - | 7.04 s |
| **1´´** | 5.20 d (1.2) | H-2´´ | 5.18 d (1.0) |
| **2´´** | 3.98 s | H-1´´e H-3´´ | 3.1-4.0 m |
| **3´´** | 3.55 dd (2.6 e 9.3) | H-2´´ e H-4´´ |  |
| **4´´** | 3.16 m | H-3´´ e H-5´´ |  |
| **5´´** | 3.37 m | H-4´´ e H-6´´ |  |
| **6´´** | 0.84 d (6.2) | H-5´´ | 0.79 d (5.3) |

Note: 1: chemical shift in ppm using TMS as internal standard; 2: multiplicity (s = singlet, d = doublet, dd = double doublet, m = multiplet); 3: coupling constant expressed in Hz.

**Table S2**: ^13^C NMR (100 MHz, DMSO-*d*_6_) data of myricetin-3-*O*-rhamnoside.

| **Position** | **δ^1^** | **δ^2^** | **(100 MHz, DMSO-*d*_6_ DEPT-135** | **HSQC^1^**  **m^3^** | **RMN ^13^C**  **(75 MHz, methanol- *d_4_*)**  ^[^[^1^](#_ENREF_1)^]^ |
| --- | --- | --- | --- | --- | --- |
| **2** | 156.4 | 156.4 | C | - | 158.3 |
| **3** | 136.5 | 135.1 | C | - | 134.5 |
| **4** | 177.8 | 178.2 | C | - | 177.9 |
| **5** | 161.3 | 161.8 | C | - | 162.9 |
| **6** | 98.7 | 98.3 | CH | 6.20 d | 99.9 |
| **7** | 164.2 | 166.4 | C | - | 165.4 |
| **8** | 93.5 | 94.0 | CH | 6.35 d | 94.3 |
| **9** | 157.5 | 158.8 | C | - | 157.5 |
| **10** | 104.1 | 104.5 | C | - | 105.0 |
| **1´** | 119.6 | 121.8 | C | - | 122.5 |
| **2´** | 107.9 | 107.9 | CH | 6.89 s | 109.5 |
| **3´** | 145.8 | 146.1 | C | - | 146.4 |
| **4´** | 134.3 | 135.2 | C | - | 137.1 |
| **5´** | 145.8 | 146.1 | C | - | 146.4 |
| **6´** | 107.9 | 107.9 | CH | 6.89 s | 109.5 |
| **1´´** | 101.9 | 109.3 | CH | 5.20 d | 102.0 |
| **2´´** | 70.0 | 75.1 | CH | 3.98 s | 70.3 |
| **3´´** | 70.4 | 71.0 | CH | 3.55 dd | 70.4 |
| **4´´** | 71.3 | 73.7 | CH | 3.16 m | 71.3 |
| **5´´** | 70.6 | 74.3 | CH | 3.37 m | 69.6 |
| **6´´** | 17.5 | 17.0 | CH_3_ | 0.84 d | 17.2 |

Note: 1: δ in ppm using TMS as internal standard; 2: theoretical δ obtained in the ChemDrawUltra program (version 10.0); 3: multiplicity (s = singlet, d = doublet, dd = double doublet, m = multiplet).

[1] M. Ceruks, P. Romoff, O. A. Fávero, J. H. G. Lago, ‘Constituintes fenólicos polares de *Schinus terebinthifolius* Raddi (Anacardiaceae)’, *Quim. Nova* **2007**, *30*, 597-599.
